# Is There a Relationship between Statin Use and Corneal Specular Microscopy and Topography Findings?

**DOI:** 10.14744/bej.2021.28190

**Published:** 2021-12-17

**Authors:** Bengi Ece Kurtul, Alparslan Kurtul, Kustharbek Ergashev

**Affiliations:** 1. Department of Ophthalmology, Mustafa Kemal University Tayfur Ata Sokmen Faculty of Medicine, Hatay, Turkey; 2. Department of Cardiology, Mustafa Kemal University Tayfur Ata Sokmen Faculty of Medicine, Hatay, Turkey; 3. Department of Ophthalmology, Aritmi Osmangazi Hospital, Bursa, Turkey

**Keywords:** Cornea, endothelial cell density specular microscopy, statin, topography

## Abstract

**Objectives:**

Information about the possible effect of statins on the human corneal endothelium is still not clear. This study was the first known investigation of the influence of statins on corneal specular microscopy (SM) and topography parameters.

**Methods:**

Thirty-four patients using a statin (atorvastatin) as hyperlipidemia treatment (Group 1) and 34 age- and sex-matched healthy subjects (Group 2) were prospectively included in the study. Demographic data and ophthalmic findings of participants were reported and analyzed. Cellular morphology was evaluated using the noncontact SM and corneal endothelial cell density (CECD) (cells/mm^2^), polymegathism (coefficient of variation in cell size [CV], %), and pleomorphism (% hexagonal cells [Hex]) data. Anterior chamber depth and volume, iridocorneal angle degree, average keratometry diopters were also measured with corneal topography.

**Results:**

The mean age was 53.03±7.23 years (range: 38–73 years) for Group 1 and 53.15±10.7 years (range: 34–80 years) for Group 2 (p=0.958). Group 1 consisted of 11 female and 23 male patients and Group 2 included 13 female and 21 male participants (p=0.798). The mean CECD density was significantly higher in Group 1 when compared with that of Group 2 (2544.34±244.76 cells/mm^2^ [range: 2126.60–3107.00 cells/mm^2^] vs 2404.53±285.46 cells/mm^2^ [range: 1839.80–2892.30 cells/mm^2^], p=0.034). There were no significant statistical differences in the CV and Hex values between the groups (p=0.450 and p=0.717, respectively). The corneal topographic measurements were also not significantly different.

**Conclusion:**

The findings of this study revealed higher CECD values in statin users. Statins may have beneficial effects on cornea morphology.

## Introduction

The corneal endothelium is a leaky monolayer at the posterior surface of the cornea (1-4). The number of corneal endothelial cells is slowly diminished from aging and this may cause fluctuating or occasionally blurred vision (3). Corneal endothelial cell density (CECD) is considered a key indicator for hydration control of the corneal stroma, which is essential for corneal transparency (1-5). Specular microscopy (SM) provides a non-invasive morphological analysis of the corneal endothelial cell layer (6). SM helps to measure the endothelial cell physiological reserve. It can be used for ocular surgical procedures and follow-up, evaluation of pharmaceutical exposure, and general health of the corneal endothelium (6).

Statins, or 3-hydroxy-3-methylglutaryl coenzyme A (HMG Co-A) reductase inhibitors, are primarily used for decreasing low-density lipoprotein (LDL) cholesterol, as well as for reducing atherosclerotic cardiovascular disease risk, and the side effects are generally minimal in most patients (7-9). Furthermore, there are anti-inflammatory, anti-apoptotic, anti-proliferative, and immunomodulatory effects of statins (also called pleiotropic effects of statins) (10). Numerous studies were shown benefits of statins in a variety of systemic diseases, including various types of cancer, inflammatory bowel disease, and rheumatoid arthritis (11-13). The possible effects of statin use in patients with age-related macular degeneration, glaucoma, cataract, and diabetic retinopathy were investigated, and the favorable effects of statins were demonstrated in these patients (14-22). The protective effect of simvastatin against ultraviolet B-induced corneal endothelial cell death was shown in cell culture (23). It was shown that lovastatin inhibits the thrombin-induced loss of barrier integrity in bovine corneal endothelium (24). However, information about the possible effect of statins on human corneal endothelium is still not clear. Herein, we aimed to investigate the influence of statins on corneal SM and topography parameters, as it has not been reported to date.

## Methods

After approval of ethical review committee of our university (2019/39), this prospective study was conducted at our hospital between April 2019 and June 2019. Written informed consent was obtained from each subject before enrollment and study was conducted in accordance with the Declaration of Helsinki. Thirty-four patients (Group 1) under the statin treatment (atorvastatin use at least 1 year) for hyperlipidemia referred to our clinic from our cardiology outpatient clinic were included in this study. The measured SM values of the patients were compared with those of the age- and sex-matched 34 healthy control subjects (Group 2) who were examined for a routine check in both clinics. The duration of statin use was also noted. All the participants underwent complete ocular examination of both eyes including best-corrected visual acuity assessment with Snellen chart, slit-lamp biomicroscopic examination of anterior and posterior segment, intraocular pressure measurement, and central corneal thickness (CCT) by non-contact tonometer. The mean CECD (cells/mm^2^), coefficient of variation of cell size (CV) %, and percentage of hexagonal cells (Hex %) were analyzed in each subject with non-contact SM (SP-3000 P, Topcon Corporation, Japan) by a single experienced examiner within the same time interval of the day (between 10 and 12 am). All measurements were performed at least 3 times using the “center” method and at least 110 cells were included in each measurement. Anterior chamber depth and volume, iridocorneal angle degree, and average keratometry diopters of patients were also screened by corneal topography (Sirius, CSO Inc., Florence, Italy). To eliminate the possible impacts of these conditions on CECD and morphology, the exclusion criteria in Group 1 subjects were; refractive error of ≥± 1.00 diopters, history of ocular surgery/trauma/retinal laser, corneal opacity or dystrophy, glaucoma, pseudoexfoliation, uveitis, use of contact lens, use of topical eye drops and diabetes mellitus, hypertension, uncontrolled dyslipidemia, using any medication except atorvastatin, and inflammatory diseases. The Group 2 subjects were healthy ones without any systemic and ocular diseases, or history of ocular surgery/trauma and drug use.

### Statistical Analysis

Statistical analysis was performed using the software SPSS (version 21.0, Chicago, IL, USA). Parametric data were uttered as mean ± standard deviation. Demographic data and clinical characteristics for cases and controls were compared using a two-sample t-test for continuous variables. Categorical data were expressed as number and percentages. To compare categorical data, the Chi-square test was used. P<0.05 was accepted as statistically important.

## Results

**Figure 1.**
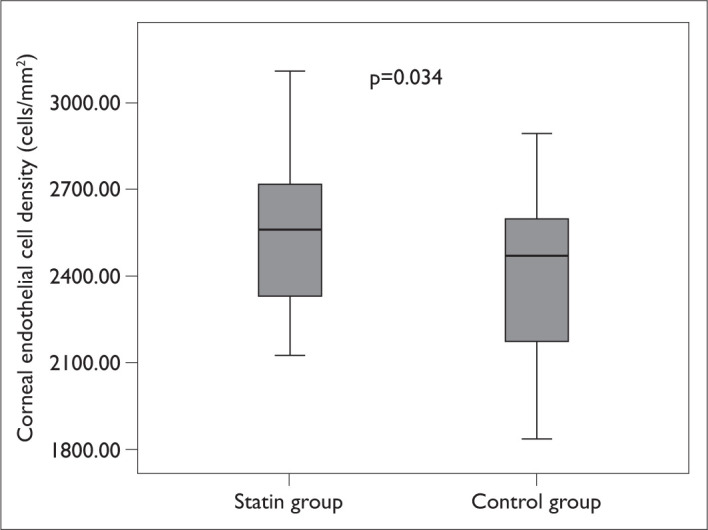
Comparison of corneal endothelial cell density values between statin and control groups.

The mean age was 53.03±7.23 (range, 38–73) years for statin users (Group 1) and 53.15±10.7 (range, 34–80) years for controls (Group 2) (p=0.958). Group 1 consisted of 11 female and 23 male patients and the Group 2 included 13 female and 21 male participants (p=0.798). Demographic characteristics of the groups are presented in Table 1. The mean CECD levels were significantly higher (Fig. 1) in Group 1 when compared to Group 2 (2544.34±244.76 [range, 2126.60–3107.00] cells/mm^2^ vs. 2404.53±285.46 [range, 1839.80–2892.30] cells/mm^2^, p=0.034). There were no statistical differences in terms of CV and Hex values between groups (p=0.450, and p=0.717, respectively). Corneal topographic measurements did not show statistically significant different between groups, either (p>0.05 for all). Clinical findings of the groups are shown in Table 2.

**Table 1.** Comparison of demographic characteristics between groups

Characteristics	Statin Group	Control Group	p-value
Number of subjects, n	34	34	
Age, years (Mean±SD)	53.03±7.23	53.15±10.7	
(min-max)	(38–73)	(34–80)	0.958
Gender, n (%)			
Female	11 (32.4)	13 (38.2)	0.798
Male	23 (67.6)	21 (61.8)	
Mean time for statin use, n (%)			
12–23 months	32 (94.1)		
	>23 months	2 (5.9)		
Body mass index (kg/m^2^)	28.7±3.5	28.3±2.8	0.285

SD: Standard deviation.

**Table 2.** Clinical, corneal specular microscopy, and topography findings between the groups

Characteristics (min-max)	Statin Group (n=34)	Control Group (n=34)	p-value
IOP (mmHg)	16.51±1.04	16.31±1.36	0.510
	(12.5–18.0)	(12.5–18.5)	
CCT (μm)	538.55±26.84	539.71±24.45	0.855
	(465–575)	(495–610)	
CECD (cells/mm^2^)	2544.34±244.76	2404.53±285.46	0.034
	(2126.60–3107.00)	(1839.80–2919.00)	
CV (%)	35.66±5.11	34.85±3.56	0.450
	(27.10–49.60)	(28.80–41.35)	
Hex (%)	52.66±7.22	53.38±9.00	0.717
	(39.00–76.50)	(38.50–77.00)	
Anterior chamber depth (mm)	3.18±0.24	3.26±0.34	0.241
	(2.52–3.56)	(2.55–4.28)	
Anterior chamber volume (mm^3^)	124.83±20.19	128.95±26.61	0.475
	(87.50–169.50)	(73.50–188.50)	
Iridocorneal angle (degree)	37.45±2.11	43.60±1.35	0.286
	(27.00–49.00)	(25.50–57.50)	
Average kerotometry (diopter)	43.72±1.91	43.60±1.32	0.777
	(39.01–48.09)	(40.99–47.07)	

IOP: Intraocular pressure; CCT: Central corneal thickness; CECD: Corneal endothelial cell density; CV: Coefficient of variation in cell size; Hex: Hexagonal cells.

## Discussion

To the best of our knowledge, this study is the first report that evaluates the SM and corneal topographic findings of patients with statin use. The results of the present study showed significant higher levels of CECD measured by the SM during long-term statin therapy in patients with hyperlipidemia.

Statins are widely used as specific inhibitor of HMG-CoA reductase and can reduce the level of LDL cholesterol (12). They have also anti-inflammatory, antiapoptotic, antiproliferative, and immunomodulatory effects (12). These are termed as so-called pleiotropic effects of statins which accompany the cholesterol-lowering effect or are seen beyond cholesterol reduction. There is growing evidence that statins may protect against the development or worsening of several ocular disorders, such as age-related macular degeneration, glaucoma, cataract, and diabetic retinopathy (14-22). On the other hand, statin therapy was found to be associated with a modestly increased risk of cataract surgery (25). Because of these contradictions, ocular therapeutic and side effects of the statins are still unclear. In a previous study conducted by Zheng et al. (26), it was demonstrated that simvastatin had a protective effect on apoptosis in diabetic rats’ retinal capillary endothelial cells, which was associated with the inhibition of mitochondrial reactive oxygen species pathway mediated by peroxisome proliferator-activated receptor gamma coactivator-1 alpha. Then, in consistent with these results, Ho et al. (23) suggested that simvastatin ameliorated ultraviolet B-induced corneal endothelial cell apoptosis through caspase-3 activity. In another study, it was shown that lovastatin inhibits the thrombin-induced loss of barrier integrity in bovine corneal endothelium (24). Furthermore, statins had been shown to reduce ocular inflammation in human models of uveitis and to prevent the development of uveitis (27). In addition, statins had protective effect in many cells type as retinal pigment epithelium by preventing oxidative stress through NADPH oxidase-dependent mechanisms (28-30). The aim of our study is to determine if statins, which are known to suppress barrier dysfunction in certain vascular endothelium (28, 29), have also similarly useful effects on the corneal endothelium. The results of the present study are consistent with findings of above-mentioned studies regarding beneficial effects of statins on the eye. Our results showed that CECD was the only significant morphologic parameter of corneal endothelial cell measurements affected by atorvastatin. There was not a significant difference between the groups regarding other parameters, such as Hex %, CV, and CCT. We think that this could happen for two reasons. First, CECD could be more sensitive than the other parameters. As a matter of fact, CECD is an important indicator for the corneal health. Another possible factor may be relatively small sample size in our study. Further prospective, randomized controlled studies with larger series of patients are needed to obtain stronger evidence.

Taken together, the present study has shown an improvement of corneal endothelial cell parameters during long-term statin therapy. Although the exact mechanism of the beneficial effect of statin use on corneal endothelial morphology is unknown, in the lights of our study results, we suggest that statin use may be related with statin-induced antioxidant effects and possible beneficial effects on corneal endothelium function, and perhaps may contribute to slowing of age-related CECD decline. We also speculate that statins may exhibit pleiotropic beneficial effects on the human corneal endothelium which seem to be mediated by the endothelium-dependent pathways. Finally, statins may have a protective effect on apoptosis (antiapoptotic effect) in human retinal capillary endothelial cells as shown before on rat model (26).

There are some limitations of our study. First, the number of study patients was relatively small because of the rigorous exclusion criteria. Our results need to be verified with a further prospective clinical trial with a larger cohort. Second, the comparison of CECD measurements in patients before and after atorvastatin treatment may be performed. Further studies are needed to investigate this special issue in more detail. Finally, the comparison between different types of statins could be investigated which could be an issue of another clinical study.

## Conclusion

SM revealed higher CECD values in patients with statin use. Statins may have beneficial effects on human cornea endothelium morphology. Several anti-inflammatory and immunomodulatory mechanisms may play crucial role this association. We suggest that ophthalmologists should take into account the history of statin use in patients when planning any ocular surgery and even clinical trials concerning cornea. Further studies are needed to confirm our results.

### Disclosures

**Ethics Committee Approval:** The present study was approved by Hatay Mustafa Kemal Univesity ethics committee (Approval nıumber: 2019/39).

**Peer-review:** Externally peer-reviewed.

**Conflict of Interest:** None declared.

**Authorship Contributions:** Involved in design and conduct of the study (BEK, AK, KE); preparation and review of the study (BEK, AK, KE); data collection (BEK, AK, KE); and statistical analysis (AK, BEK).
